# The Effect of Teachers’ Self-Efficacy and Creativity on English as a Foreign Language Learners’ Academic Achievement

**DOI:** 10.3389/fpsyg.2022.872147

**Published:** 2022-04-12

**Authors:** Yuanyuan Ma

**Affiliations:** Department of Elementary Education, Jiaozuo Normal College, Jiaozuo, China

**Keywords:** teacher creativity, teacher self-efficacy, academic achievement, EFL learners, education

## Abstract

Since learners’ academic achievement is the ultimate goal of any system of education, multitudes of studies have been conducted on this variable and its determinants. That is, several scholars have explored the effects of student-related and teacher-related factors on learners’ academic achievement. Notwithstanding, a few researchers have investigated the effects of teachers’ self-efficacy and creativity on learners’ academic achievement. Furthermore, no study has reviewed the role of these teacher-related factors in learners’ achievement. Therefore, the present study seeks to bridge the lacunas by delving into the influence of teachers’ self-efficacy and creativity on English as a Foreign Language (EFL) learners’ academic achievement. The beneficial effects of self-efficacy beliefs and creative teaching were outlined through the existing evidence. This seems to be illuminative for EFL teachers. Given the positive consequences of teachers’ self-efficacy for students’ achievement, EFL teachers should believe in themselves and their abilities to lead their students to academic success. Additionally, with regard to the positive impact of teachers’ creativity on learners’ achievement, EFL teachers are expected to teach the course content creatively.

## Introduction

In any instructional-learning context, learners’ academic achievement is the primary focus of policymakers, educational administrators, and instructors, and English as a Foreign Language (EFL) contexts are not an exception by any means. Academic achievement is “the outcome of learning, which is typically measured by classroom grades, classroom assessments, and external achievement tests” (Gajda et al., 2017, p. 270). Put simply, academic achievement pertains to the extent to which learners have effectively acquired and mastered course content (Ho and Hau, 2008). In the language education domain, academic achievement deals with how well language learners have acquired a new language (Cho et al., 2018). Owing to the fact that language learners’ personal qualities and characteristics may serve an influential role in enhancing their academic achievement (Komarraju et al., 2009; Hakimi et al., 2011), numerous inquiries have been done to uncover the effects of student-related variables, including motivation, self-concept, and self-efficacy on language learners’ academic achievement (e.g., Othman and Leng, 2011; McInerney et al., 2012; Chen et al., 2013; Soodmand Afshar et al., 2014; Wang, 2015; Li and Lynch, 2016; Li, 2020, to cite a few). Given the central position of teachers in classroom contexts, several research studies have also been conducted on the impact of teacher-related factors on language learners’ academic achievement (Hofferber et al., 2014; Kola and Sunday, 2015; Rahimi and Karkami, 2015; Klusmann et al., 2016; Kheirzadeh and Sistani, 2018; Kim and Seo, 2018; Ma et al., 2018, to cite a few). Yet, the effects of teachers’ self-efficacy and creativity on language learners’ academic achievement have received relatively little attention (Gowrie and Ramdass, 2014; Kim and Seo, 2018).

The term “self-efficacy” generally refers to an individual’s belief in his or her own ability to successfully carry out a given action (Bandura, 1997). Applied to the educational context, teacher self-efficacy pertains to “a teacher’s judgment of his/her capabilities to bring about desired outcomes of student engagement and learning, even among those students who may be difficult or unmotivated” (Tschannen-Moran and Hoy, 2001, p. 784). Put differently, teacher self-efficacy deals with teachers’ beliefs in their capacity and ability to fulfill the educational responsibilities they are in charge of (Klassen and Chiu, 2010). As Klassen et al. (2014) submitted, teachers who strongly believe in themselves and their instructional abilities are able to: (a) utilize alternate approaches and techniques when the expected outcomes are not attained and (b) manage a difficult condition by altering the conditions’ cognitive and emotional processes. Teachers with high levels of self-efficacy, according to Gibbs and Powell (2012), are capable of increasing their students’ academic achievement as well. That is, teachers’ self-efficacy is believed to favorably influence student academic achievement (Han and Wang, 2021).

Teacher creativity as another influential factor in learners’ academic achievement pertains to “the utilization of imaginative approaches to make learning more interesting” (Jeffrey and Craft, 2004, p. 78). In another definition, Nunan (2013) referred to this concept as “the recombination of familiar elements into new and previously unrehearsed forms” (p. 70). More specifically, language teachers’ creativity deals with their ability to offer some opportunities to their pupils to use the language they have been educated in novel and innovative ways (Richards, 2013). Creative teaching is thought to improve language learners’ interest, academic motivation, and academic achievement (Baghaei and Riasati, 2015; Xerri and Vassallo, 2016).

Despite the prominence of teachers’ self-efficacy and creativity in improving learners’ academic achievement (Gibbs and Powell, 2012; Xerri and Vassallo, 2016), the effects of these teacher-related variables on learners’ academic achievement have not been widely studied (Gowrie and Ramdass, 2014; Kim and Seo, 2018). Moreover, only a few researchers have probed the impact of teachers’ creativity and self-efficacy on EFL learners’ academic achievement (Mojavezi and Tamiz, 2012; Rashidi and Moghadam, 2014). Additionally, no review study has been conducted on this subject to highlight the positive consequences of teachers’ self-efficacy and creativity for EFL learners’ academic achievement. This review study attempts to bridge the gaps by illustrating the function of teachers’ creativity and self-efficacy in promoting EFL learners’ academic achievement.

### Teacher Self-Efficacy

The construct of self-efficacy generally pertains to one’s beliefs, thoughts, and perceptions about his/her personal abilities and capabilities (Bandura, 1994). Likewise, teacher self-efficacy deals with how individual teachers perceive themselves, their instructional competence, and their professional skills (Klassen et al., 2010). Skaalvik and Skaalvik (2010) defined teacher self-efficacy as “an individual teacher’s beliefs in his or her ability to plan, organize, and carry out activities that are required to attain educational goals” (cited in Sarfo et al., 2015, p. 20). Further, Tschannen-Moran and Johnson (2011) asserted that teachers’ self-efficacy is not just a personal evaluation of professional capabilities, but rather a belief about what they can do in diverse situations. As mentioned by Tschannen-Moran and Hoy (2001), the construct of teacher self-efficacy entails three broad dimensions, including “*efficacy for instructional strategies*,” “*efficacy for classroom management*,” and “*efficacy for student engagement*.” Accordingly, teachers with strong self-efficacy beliefs are able to engage students in learning tasks, utilize effective teaching techniques, and control the classroom atmosphere (Fathi et al., 2020).

### Teacher Creativity

The concept of creativity has been theoretically defined as “the interaction among aptitude, process, and environment by which an individual or group produces a perceptual product that is both novel and useful” (Plucker et al., 2004, p. 91). To put it in a nutshell, creativity is the use of imagination, inspiration, and innovative ideas to achieve a certain goal (Cheng, 2010). Applied to the educational context, teacher creativity pertains to individual teacher’s use of innovative approaches, methods, and techniques to improve students’ learning outcomes (Ghanizadeh and Jahedizadeh, 2016). As Lou et al. (2012) noted, creative teaching enables teachers to become aware of their full potential as educators, but only if they have mastered the subject matter themselves. Creative teaching is also believed to positively influence students’ academic attainment, academic performance, and learning outcomes (Richards, 2013).

### The Effect of Teachers’ Self-Efficacy and Creativity on EFL Learners’ Academic Achievement

To explain the value of teachers’ personal traits in promoting English language learners’ academic achievement, Akbari and Allvar (2010) postulated that teachers’ self-efficacy beliefs inspire them to enhance their professional attempts. Put simply, self-efficacy beliefs act as a motivator, encouraging teachers to give their all in the classrooms. This, in turn, contributes to increased student achievement. In a similar vein, Gibbs and Powell (2012) also stated that instructors with strong self-efficacy beliefs invest more effort in teaching course content, which enables them to improve their learners’ language achievement. Besides, to illustrate the role of teacher creativity in learners’ academic achievement, Papa (2015) submitted that creative teachers commonly employ innovative methods, strategies, and materials that make the learning process more enjoyable. An enjoyable learning atmosphere will drive students toward higher academic outcomes (Dewaele et al., 2019; Wang et al., 2021).

## Empirical Studies

As mentioned earlier, despite the value of teacher self-efficacy and creativity in learners’ academic achievement, few investigations have been performed to explore the effects of these teacher-related factors on EFL learners’ academic achievement (Mojavezi and Tamiz, 2012; Baghaei and Riasati, 2015; Kim and Seo, 2018). Mojavezi and Tamiz (2012), for example, delved into the role of teacher self-efficacy in EFL learners’ academic achievement. To do this, 80 EFL teachers and 150 EFL students were selected from different high schools in Iran. To collect data, two close-ended scales (i.e., teacher self-efficacy scale and student achievement scale) were distributed among participants. The analysis of participants’ answers demonstrated that teachers’ self-efficacy beliefs can positively affect EFL learners’ academic achievement. Moreover, Baghaei and Riasati (2015) examined the influence of teacher creativity on EFL learners’ academic achievement. In doing so, six EFL teachers and 81 EFL learners were asked to respond to two different questionnaires. The responses were analyzed using Pearson correlation and one-way ANOVA. The results indicated that EFL learners’ academic achievement can be favorably affected by teachers’ creativity.

## Implications

So far, the variables of teacher creativity, self-efficacy, and learner academic achievement were described. Moreover, in light of available evidence, the effects of teacher self-efficacy and creativity on EFL learners’ academic achievement were illustrated. Taking the theoretical and empirical evidence into consideration, one can logically infer that both self-efficacy beliefs and creative teaching positively influence EFL learners’ academic achievement. This finding seems to be beneficial and enlightening for EFL teachers. With respect to the positive consequences of teachers’ self-efficacy for students’ achievement, EFL teachers need to believe in themselves and their abilities to lead their students to academic success. Further, considering the positive impact of teachers’ creativity on learners’ achievement, EFL teachers are required to make use of creative teaching in their classes. Due to the scarcity of research on the role of teachers’ creativity and self-efficacy in learners’ academic achievement, further empirical studies are thus needed.

## Author Contributions

The author confirms being the sole contributor of this work and has approved it for publication.

## Conflict of Interest

The author declares that the research was conducted in the absence of any commercial or financial relationships that could be construed as a potential conflict of interest.

## Publisher’s Note

All claims expressed in this article are solely those of the authors and do not necessarily represent those of their affiliated organizations, or those of the publisher, the editors and the reviewers. Any product that may be evaluated in this article, or claim that may be made by its manufacturer, is not guaranteed or endorsed by the publisher.
